# Making social sciences more scientific: Literature review by structured data

**DOI:** 10.1016/j.mex.2020.100818

**Published:** 2020-02-20

**Authors:** Vuong Quan-Hoang, Le Anh-Vinh, La Viet-Phuong, Hoang Phuong-Hanh, Ho Manh-Toan

**Affiliations:** a Center for Interdisciplinary Social Research, Phenikaa University, Hanoi 10000, Vietnam; b Faculty of Economics and Finance, Phenikaa University, Hanoi 10000, Vietnam; c The Vietnam National Institute of Educational Sciences, Hanoi 10000, Vietnam; d A.I. for Social Data Lab, Vuong & Associates, 3/161 Thinh Quang, Dong Da District, Hanoi, 100000, Vietnam

**Keywords:** Structured data, Literature review, Database, Vietnam, SSHPA, Social Sciences and Humanities Peer Awards, NAFOSTED, National Foundation for Science & Technology Development, SDA, SSHPA Data Analysis

## Abstract

The paper proposes a new method for conducting a literature review by structured data of more than 2200 scientific articles and 1300 researchers on SSHPA (Social Sciences and Humanities Peer Awards), an open database of Vietnamese social scientists’ scientific productivity. Based on the logical structure of SSHPA, the authors create a specialized database for the literature review: SDA (SSHPA Data Analysis). Combining expert's caliber and computational algorithms, SDA is expected to offer an immensely efficient and analytical based method of scanning data, hence ameliorating the traditional approach to conducting a literature review.

• A specialized database for literature review is created using the scientific articles and author profiles from SSHPA, an open database of Vietnamese social scientists’ productivity.
• The review database assigns values of topics or methodological attributes to articles sourced from SSHPA.
• Then, the authors can query comprehensive data tables, graphs, or diagrams to use for literature review.

## Graphical abstract

**Figure:**
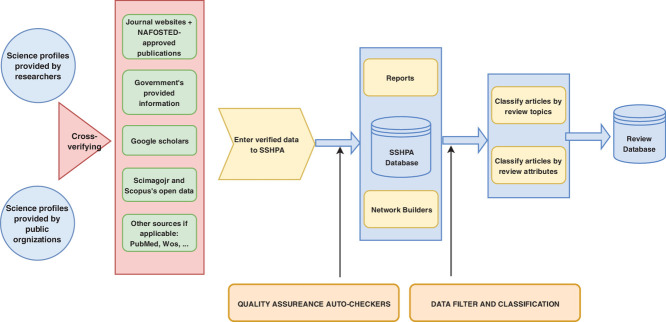

**Table:** Specification Table

Subject Area:	*Social Sciences*
More specific subject area:	*Entrepreneurship, Vietnam Social Sciences, and Humanities*
Method name:	*A method of literature review by structured data*
Name and reference of the original method:	
Resource availability	https://sshpa.com/

## Method details

The literature review examines important findings and shows potential directions, which presents discussions and analysis of existing knowledge concisely and systematically. However, doing a literature review is a time-consuming and labor-intensive work that requires the investigation and critical analysis of hundreds or more articles, and this often leads to information overload [1]. Moreover, there is the question of ‘enough’: is the number of articles enough? Is the scope and coverage of knowledge wide enough? Another issue is the scattering numbers of papers among many scientific databases, especially when reviewing a specific field such as social sciences and humanities [2].

In order to address this problem, the authors propose a new method: a literature review by structured data. The method is developed based on the power of a database on the scientific productivity of Vietnamese social sciences and humanities researchers – SSHPA (Social Sciences and Humanities Peer Awards). We extract and customize data from SSHPA with extra information to look for deeper insights.

In the article, we will firstly explain the system architecture and data structure of the SSHPA database, and its expansion: SDA Review Database. Then, the construction and quality assurance process of the SDA (SSHPA Data Analysis) Review Database are described in sections II and III. Finally, the review capacity and research potential of the SDA Review Database are discussed using examples from a review of the entrepreneurship subfield.

## SSHPA database

The data for the literature review method comes from our database called SSHPA (https://sshpa.com/). The system was built to monitor the scientific productivity of Vietnamese social sciences and humanities researchers, and datasets from the system were the canon of 6 scientific publications on the topic [3], [4], [5], [6], [7], [8]. As of January 23, 2020, the database has recorded up to 2002 Vietnamese researchers and 3140 scientific articles from 2008 until now, and the numbers are still growing. To validate the potential of the recorded data for reviewing the literature, we will first explain the methodology and logic of the assembly of SSHPA.

### The system architecture of SSHPA

**Fig. 1:**
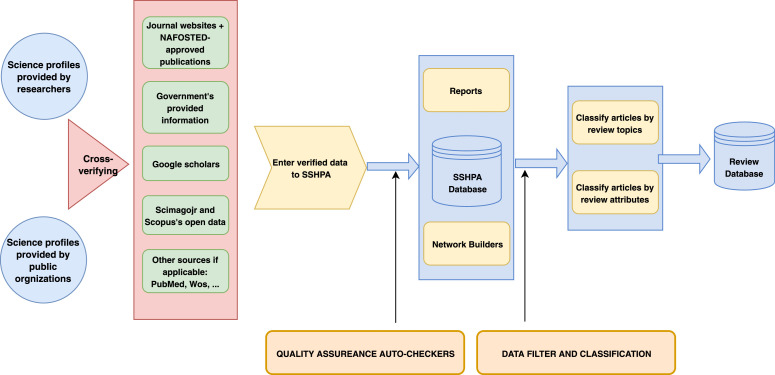
Workflow of SSHPA and SDA database. Recreated from Fig. 3 in Vuong et al. [8].

The construction of the system involves three major stages; all visualized in Fig. 1. The first stage required the collection of Vietnamese nationality scientists in the field of Social Sciences and Humanities who are affiliated with an organization in the country from their public science profiles. These researchers also had to have published at least one paper in Scopus-indexed scientific journals, using data collected in Vietnam or covering Vietnam related topics in the field from 2008 to now. The specific period of time was targeted because 2008 marked the foundation of the National Foundation for Science & Technology Development (NAFOSTED), which, with its open assessment approach based on individual productivity and higher standards imposed on international publications, has boosted research quality in Vietnam for both natural and social sciences.

After cross verification with other open access sources including those from the government's, NAFOSTED's, Scopus's open-access data and websites of other scientific journals, the manually verified data were then entered into the SSHPA database by data collectors and went through the second stage: automated quality assurance and control. The purpose of this stage was to filter out invalid or bad data, i.e., articles or authors that are not fitted into the proposed criteria or include inaccurate information, by using error reports, visualization of data such as networks generated by the system. The credibility of the system has been enhanced by the direct cooperation from the SSHPA-indexed Vietnamese researchers in the verification process of their profiles.

Finally, the system architecture employs a three-level authorization as the last stage to minimize and detect human errors timely: collectors, supervisors, and admins. By assigning specific authority to each level, the system has been developed to optimize the accuracy and reliability of the entered data [8]. Finally, further data analysis is conducted in the SDA review database, which will be explained in detail in section II.

### SSHPA data structure

The rigorous system of SSHPA utilizes both human analysis and machine algorithms to verify and clean data. It is, therefore, able to avoid many problems such as name duplication or slow data updates.

**Fig. 2:**
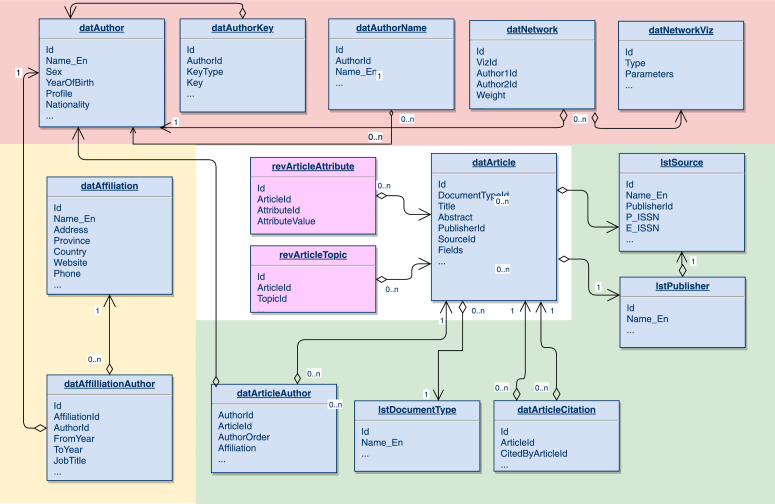
SSHPA's and SDA's data structure diagram. Recreated from Fig. 4 in Vuong et al. [8].

Input data in the database system were categorized into four types as shown in Fig. 2, authors and their networks data (pink block), information from the sources (green block), publishers and articles and data about authors’ affiliation (yellow block), which are all connected through Article as a fundamental unit. This is because the title of an article is long enough to eliminate duplications while data stored in DatArticle box, including title, publisher ID, journal ID, etc. origin from other boxes containing information about the publishers, the sources, information types, etc. Finally, SDA data represented by revArticleAttribute and revArticleTopic boxes were added to the structure as an expansion of the SSHPA system.

### An efficient method to construct a literature review

The client-serving architecture of the SSHPA database system offers an automated generation of network data and descriptive statistics. This is a crucial tool when searching for information for a literature review as it helps to specify key articles and authors promptly. The advanced search options yield immediate information about authors publishing the most articles concerning searched topics, all the related work done by the same author, or journals with the most relevant articles. For instance, key authors in a particular field of study could be identified by the size of dots in the collaboration network of researchers in the field with bigger dots representing authors with a higher number of publications within a specific period. The process of literature research could potentially be liberated from manual labor also by the generation of datasets and reports of various forms to serve data analysis purposes. The system, therefore, will save scientists a considerable amount of time spent searching for sources in the first stage of conducting a literature review.

According to Pho and Tran [9], two of the biggest challenges faced by Vietnamese researchers when publishing internationally are lack of time and funding. An open-access database that allows automated visualization of network data and datasets is particularly meaningful to improve the productivity of scientific research in Vietnam, specifically in the field of Social Sciences and Humanities.

**Fig. 3:**
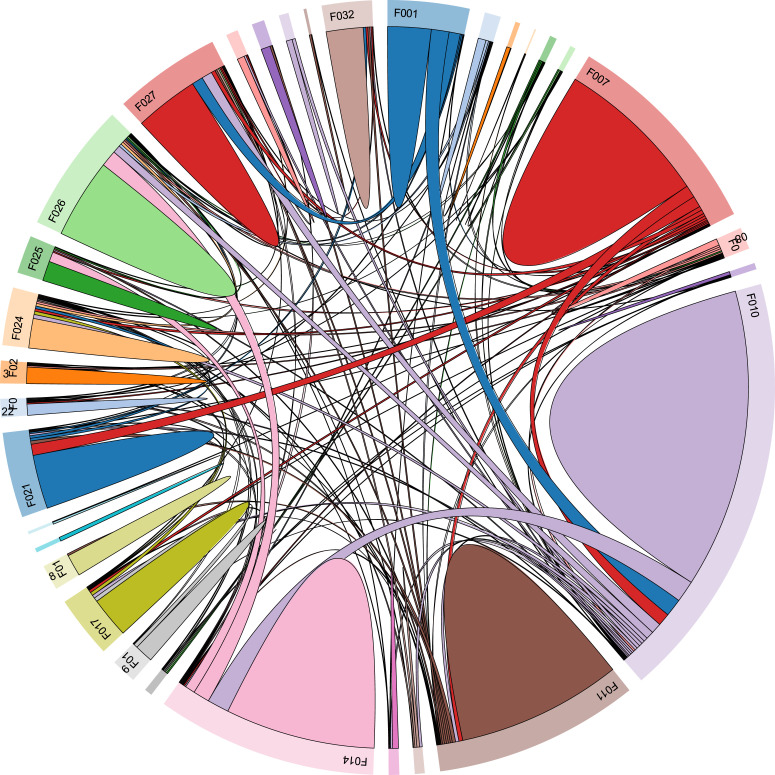
Example of a chord diagram displaying connections between 28 Social Sciences and Humanities fields in SSHPA.

Another review-serving function of the system is the visualization of the relationship among articles from various fields of study. It makes clear to researchers which areas of research are closely relevant, hence, offering an interdisciplinary background of the topic interested, as an example in Fig. 3. Developing a broad understanding of the area is necessary to establish analyses in a literature review and also enable researchers to examine their topic in a larger context where new contributions might result from their work [10,11]. Furthermore, interdisciplinary collaboration has increasingly become popular as the need to integrate various research fields to fully answer raised questions or allow the application of findings in a specific topic [12]. For example, to thoroughly examine the concept of cultural additivity, Vuong and his collaborators had to review relevant concepts from various fields such as hybridity, creolization, and syncretism from anthropological, religious, as well as cultural contexts [13].

## SDA review database

Despite the SSHPA system structure's detailed information concerning scientific articles and potential to generate results suitable for literature review, some shortcomings require system modification. Firstly, the system was built to investigate the scientific productivity of Vietnamese researchers. Thus, the information is optimized exclusively for this purpose. Moreover, the logical structure of SSHPA was proved to be efficient; therefore, any expansion might interfere with the current logical structure and require tremendous technical effort. Finally, many important working papers and reports are missing from the SSHPA database because the system only covers Scopus-or-ISI-indexed papers from 2008 until now. To address the problems, we decided to create an expansion of the SSHPA database called the SDA Review Database (http://sda.sshpa.com/). SDA anchors to the vast amount of data from SSHPA yet has its customized variables and tools to explore the data. Using an example of the review process of the entrepreneurship subfield on SDA, we expect to exploit the SSHPA data peculiarly for literature review purposes. The data is available as supplementary materials, and the full-length manuscript can be read in [14].

SSHPA consists of 28 Social Sciences and Humanities fields including such disciplines as Agriculture, Anthropology, Applied Math, Archeology, Architecture, Art, Asian Studies, Business, Cultural Studies, Demography, Economics, Education, Forestry, Geography, Health Care, History, International Relations, Law, Literature, Logistics, Management, Media/Journalism, Philosophy, Political Science, Psychology, Sociology, Statistics, Tourism, and Urban Studies, which constitute a small element of Article unit: field. A literature review requires breaking down a field into subfields and topics. For instance, entrepreneurship belongs to the larger fields of economics, business, and management, but there are also various smaller topics concerning entrepreneurship, such as cultural influences or economic efficiency. Two unique data unit of SSHPA shown in Fig. 2: revArticleAttribute and revArticleTopic, represent these interconnections among articles’ attributes and topics.

**Fig. 4:**
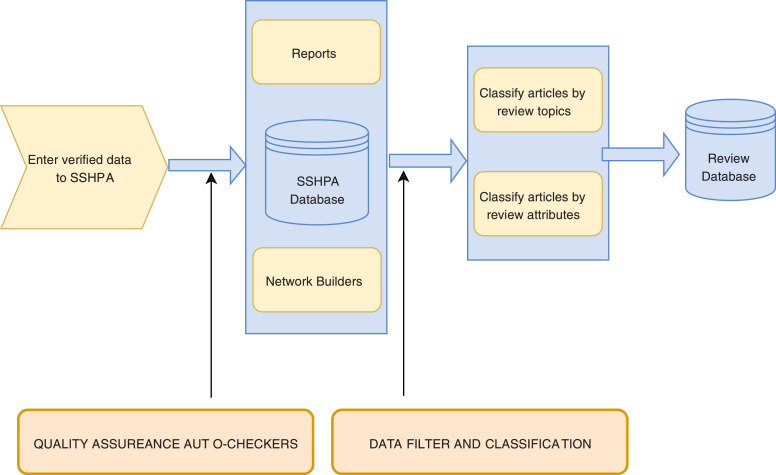
Workflow of SDA database.

**Fig. 5:**
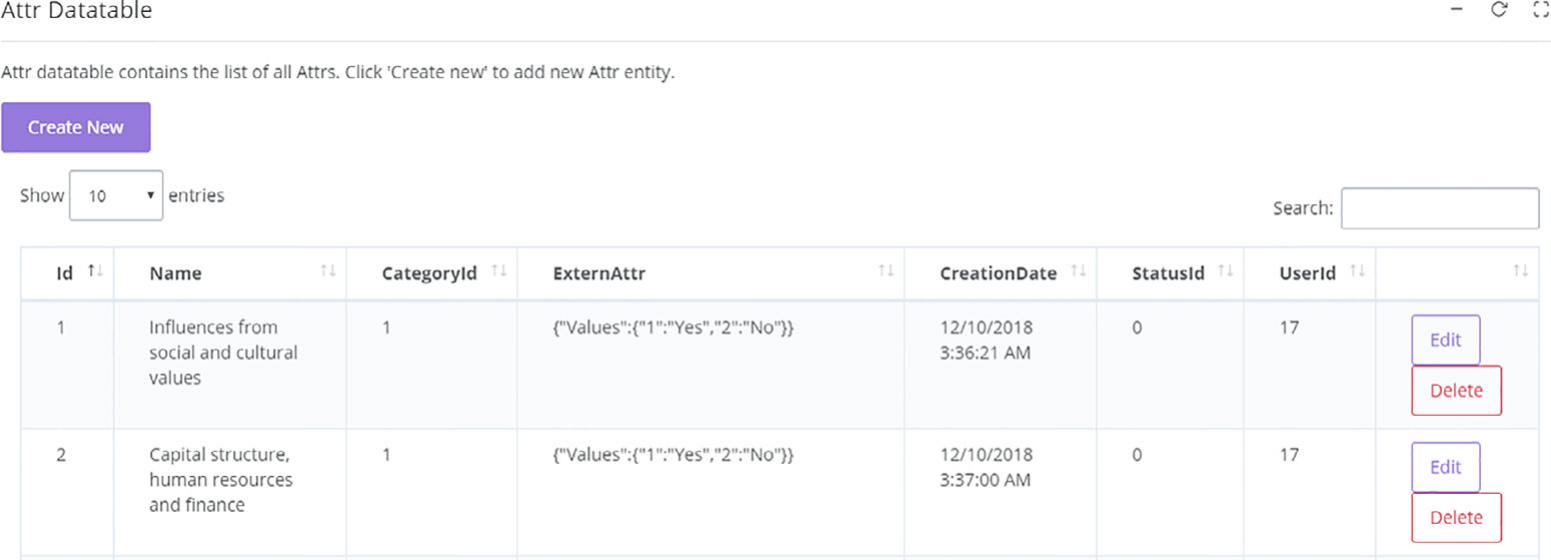
Attribute Datatable in SDA.

Similar to SSHPA, SDA is a semi-automated system utilizing both human knowledge and computational power in its workflow, as presented in Fig. 4. Human expertise is especially important in designing information architecture, data filter and classification, and quality assurance. Before entering the data from SSHPA to SDA, we must identify the review attributes. Research topics are an essential aspect of the literature review, so we built the review attributes to highlight important ones. In this stage, a group of authors will scan the literature to propose a list of significant topics. The list will be reviewed by experts in the field before it can be finalized. Then, we created attributes and their values on the SDA system: an attribute that indicates topics will have either “yes” or “no” values (Fig. 5). The creation of attributes is, on the other hand, flexible and allows customization based on the specific requirements. For instance, a variable that indicates methodological aspects will have detail categorical values such as “qualitative,” “quantitative,” or “review.” That process requires expertise in defining and choosing review attributes when designing the system.

**Fig. 6:**
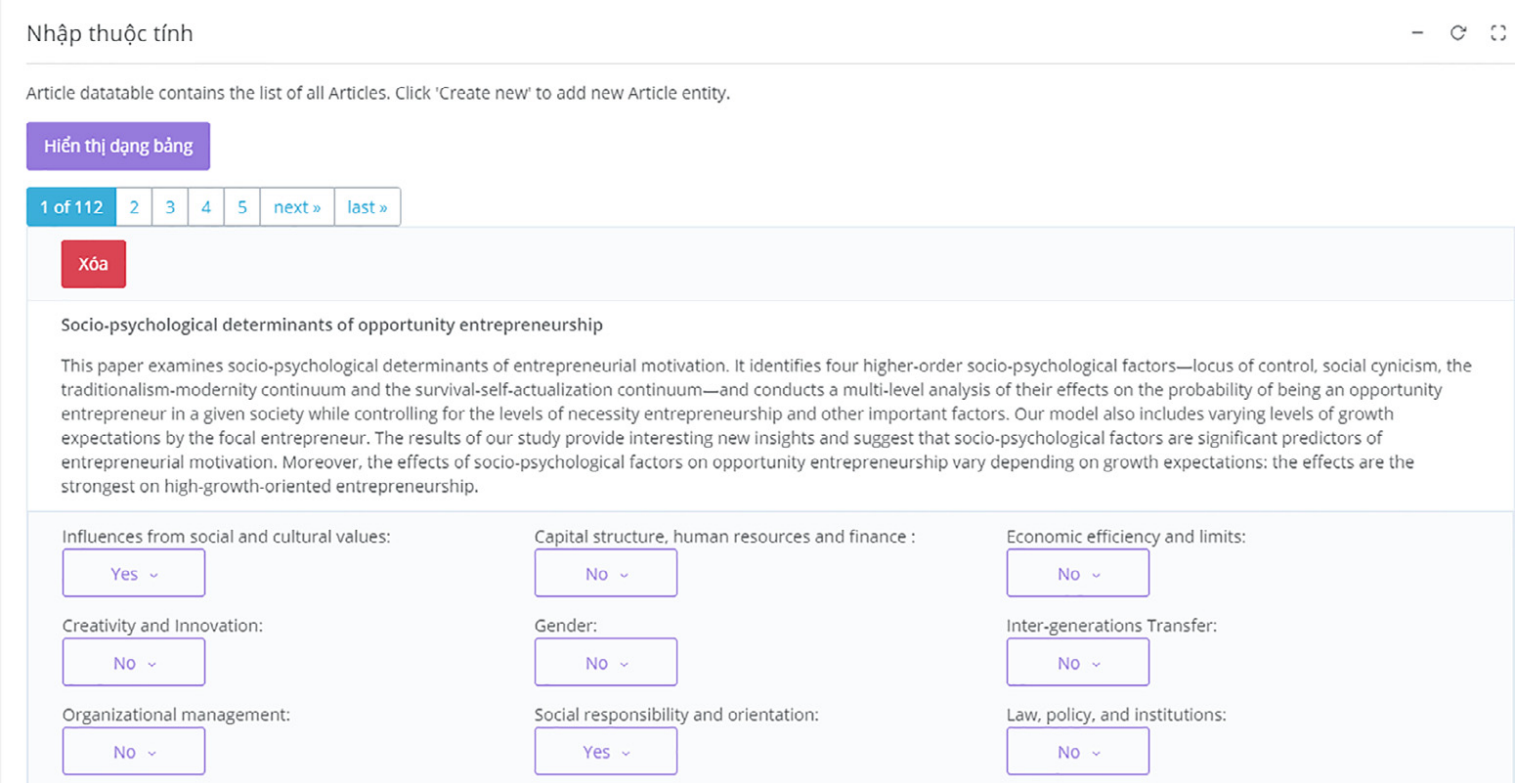
Attribute Input in SDA.

Next, we import the articles from SSHPA to SDA and start assigning values to the review attributes of each of the articles (Fig. 6). The articles from SSHPA are searched for using multiple keywords related to the review subfields. In the case of entrepreneurship subfield, keywords such as entrepreneurship, entrepreneur, entrepreneurial firms, small and medium enterprises, small business, startup, micro firms, and microfinance are used for searching.

When the data is completed, the team of authors will examine the data, data tables, and visualizations. The main purpose of the literature review is to identify research trends and patterns of a particular topic to set forth new challenges for the field. Thus, SDA is capable of exporting data tables in CSV format (Supplementary material) for statistical analysis and instantly generating data visualization. If the data project shows an abnormal pattern, experts’ knowledge is needed to determine whether the data is accurate or not.

The computational power helps SDA exploit the resources of the SSHPA database; however, the SSHPA database has restricted scope of coverage: (1) Scopus-or-ISI-indexed papers from 2008 until now, and (2) papers by Vietnamese authors only. As mentioned above, these criteria lead to the exclusion of scientific articles from before 2008, and important working papers or reports. We were reluctant to tackle this problem because it would either interfere with the SSHPA data structure or create an unnecessary workload for the system. Moreover, when considering the topic of entrepreneurship research in Vietnam, we were able to collect a reasonable number of 112 articles from 2008. Therefore, we proposed that the collected data and the out-of-scope papers could be discussed in the introduction section to set the context for the literature review.

## Quality assurance

The SSHPA database was designed to eliminate problems that Scopus or ISI Web of Science faces: data duplication and slow update. If any of these occur to author names, article titles, or affiliations, the system will be able to generate a report informing the admins of the duplicated or missing data [8]. The quality assurance process of SSHPA secures the reliability of data for the literature review purpose in SDA. In the SDA database, quality assurance relies heavily on the expertise when designing the study and when reviewing the data tables and visualization rendered by the system. The following boxes are SQL code for extracting Entrepreneurship articles and authors by year: (Boxes 1 and 2).

Box 1 **SQL code for extracting Entrepreneurship articles by year.**

select count(ar.Id) as ArticleCount, ar.PublishYear from datArticle as ar

where ar.PublishYear >= 2008 and ar.PublishYear <= 2018

and exists (select 1 from revArticleCategory as revArCat inner join revCategory as revCat on revArCat.CategoryId=revCat.Id

where revArCat.ArticleId=ar.Id and revCat.Name='Entrepreneurship')

group by ar.PublishYear

Box 2 **SQL code for extracting Entrepreneurship authors by year.**

select count(*) as ArticleCount, aa.Name_En, cc.PublishYear

from datAuthor as aa inner join datArticleAuthor as bb on aa.Id=bb.AuthorId inner join datArticle as cc on bb.ArticleId=cc.Id

where cc.PublishYear >= 2008 and cc.PublishYear <= 2018

and exists (select 1 from revArticleCategory as revArCat inner join revCategory as revCat on revArCat.CategoryId=revCat.Id

where revArCat.ArticleId=cc.Id and revCat.Name='Entrepreneurship')

group by aa.Name_En, cc.PublishYear

**Fig. 7:**
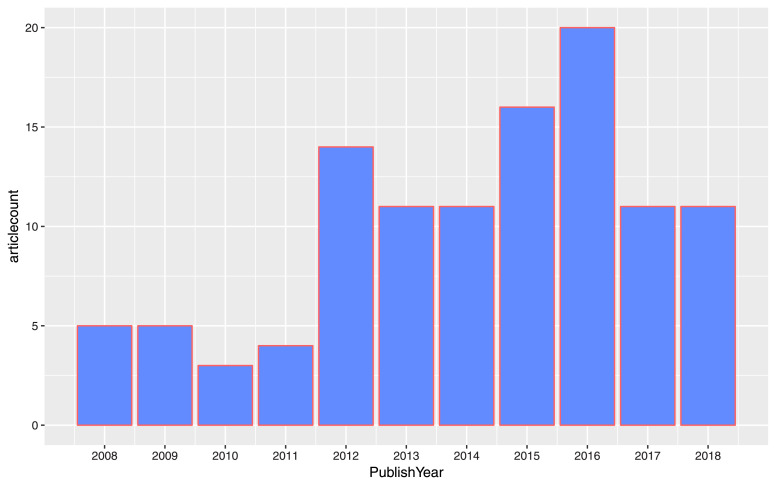
Example of data visualization of the total output in the entrepreneurship research in Vietnam.

**Fig. 8:**
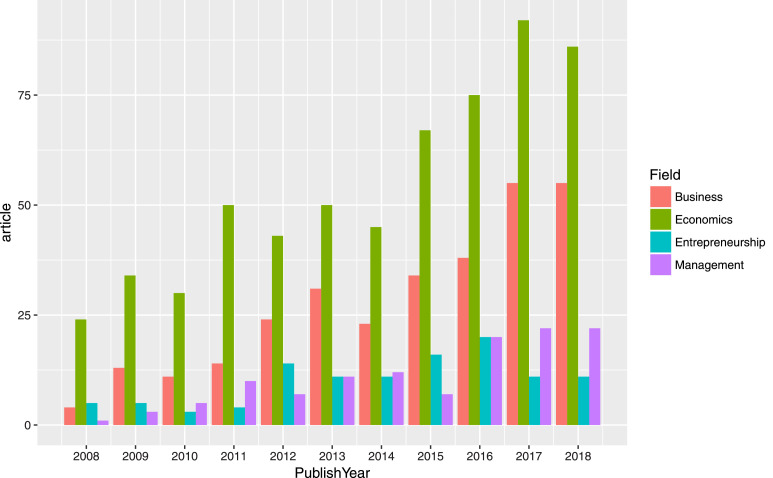
Example of output of SDA subfield (Entrepreneurship) against SSHPA fields (Business, Economics, and Management).

The SQL code is to extract data tables on SDA and ensure the process will run smoothly. Based on the data tables, we can examine the data using statistical tools. Furthermore, quality assurance requires expertise to investigate data output. Through the process, the authors perceive not only problems but also stories told by structured data. For instance, Fig. 7 illustrates the article output of entrepreneurship research in Vietnam from 2008 to 2018. Besides the overall development pattern of productivity, highlights such as the 2010 striking fall in the number of publications could also be noted, suggesting possible data disruption. If one refers to Fig. 8, the same downfall could be observed in the fields of Economics, Business, and Management. This, however, might imply an interesting underlying story rather than a problem.

The system structure and human expertise ensure the data are reliable and ready for conducting research. In the next part, we would like to present some preliminary results to illustrate the capacity of the system.

## Review capacity and research potential

The SDA review database generates multiple data tables, graphs, and diagrams. In Table 1, we have data of 12 Vietnamese authors in the top 10% of Vietnamese researchers in the field of Entrepreneurship. The system automatically informs us that these authors produced 53,13% of the total research productivity in the period 2008–2018. The system allows querying not only the top 10% but also necessary percentiles.

**Table 1:** Example of the top 10% of Vietnamese researchers in the field of Entrepreneurship.

SDA ID	SSHPA ID	Number of article
5	vm.3	13
6472	vf.2081	9
30	vf.25	7
10	vm.12	6
22	vm.16	6
84	vm.79	5
42	vf.37	4
6489	vm.2087	4
6516	vm.2104	3
480	vm.438	3
120	vm.115	3
81	vm.76	3

**Fig. 9:**
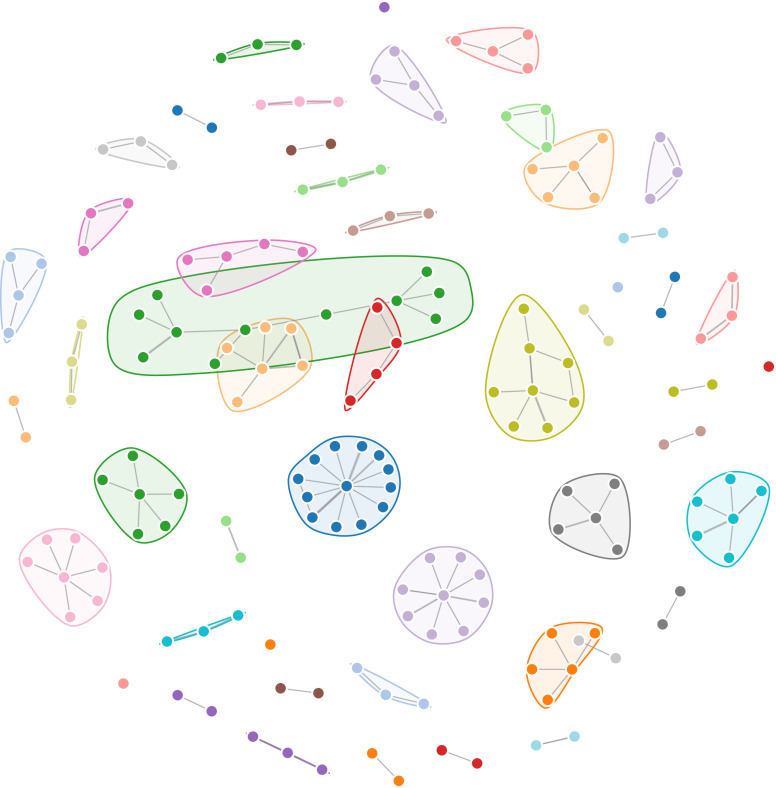
Example of a network of author groups in Entrepreneurship research in Vietnam. If authors have collaborated on at least one publication, they are circled out to indicate a research group.

**Fig. 10:**
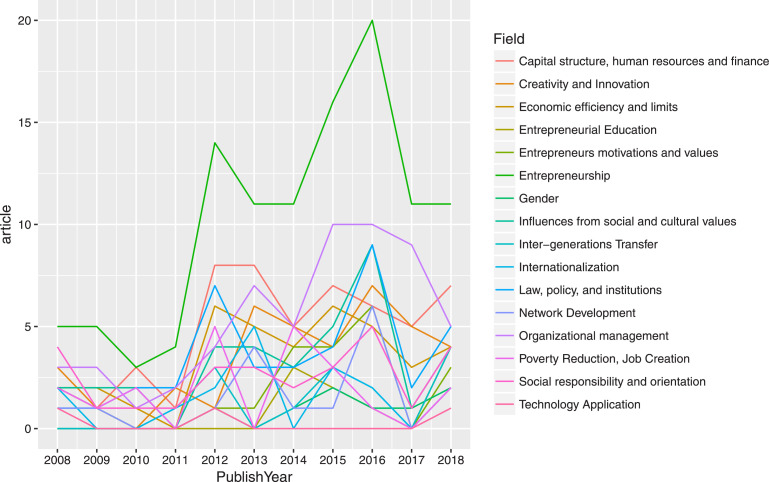
Example of article output in each topic in entrepreneurship research in Vietnam.

Another strength of SDA is the ability to generate graphs and diagrams instantly. For instance, Fig. 9 shows the network of groups in Entrepreneurship research. It could be learned from the diagram that collaboration among 2–3 authors as a group is most prevalent in the area of Entrepreneurship. Connecting with article output and topics shown in Fig. 10, it could reveal intriguing insights about the research behavior of Vietnamese scientists. Previous research [7] employed similar tools to examine co-authorship patterns of Vietnamese researchers from 2008 to 2017, which revealed that the sharing of knowledge and expertise in the Vietnamese social sciences network is not consummate, causing waste of resources and low productivity.

**Fig. 11:**
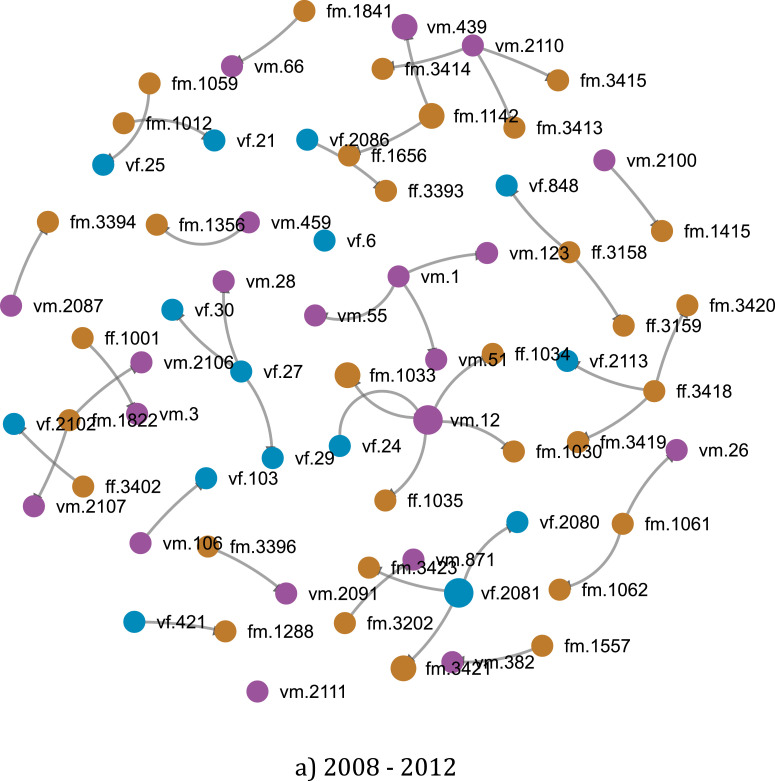

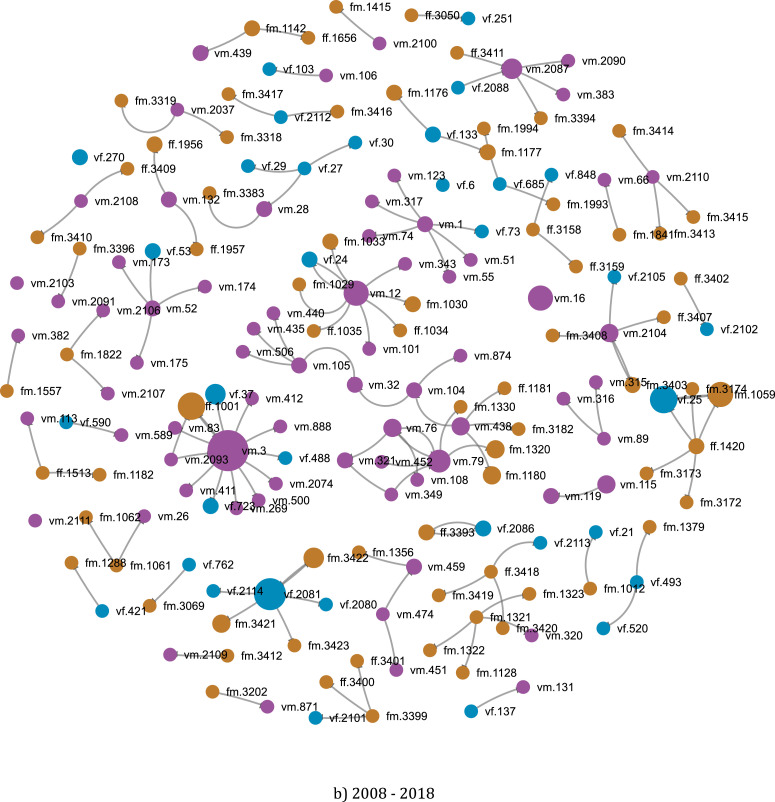
Example of a network of collaborations of Entrepreneurship research in Vietnam in two periods: (a) 2008–2012; and (b) 2008–2018. Each dot represents a researcher, and the size of the dot shows the productivity of the researcher. Males are colored (by computer code) with purple, female as blue, and foreign researchers as orange.

Also interested in the properties of the network of scientists in Vietnam, Vuong et al. [6] sought to uncover the evolution patterns of scientific groups over time. The method they employed was to obtain various sets of data representing the circle of the collaboration of particular researchers in different chronological periods, as modeled in Fig. 11. Information about how the collaboration network of an author has evolved over time, as well as his/her output growth rates, could be deducted using this method. The same algorithm was also performed to render a visual map of Vietnamese overall connection and productivity from which another study [4] proposed a new approach to define academic productivity other than article count. The researchers were also able to obtain statistical data concerning demographic factors as potential influences on the proposed count of publications using one of the automated properties of the SSHPA database system. The paramount data presented in these studies have justified a novel yet vigorous logic to assemble and process data a research review. The rendered overall picture of collaboration and trends in research over periods combined with the structured data of SDA have proved valuable in bringing substantial power to research validity and reliability.

## Making social sciences more scientific

Developers of SSHPA aimed to exploit the availability of free sources of data to construct a versatile database system with minimum cost [8,15]. The system's logic and technical monitoring have been validated to have liberated research effort spent systemizing knowledge and scientific findings. On that basis, SDA, as an expansion of SSHPA to upgrade methodologies on literature review, which combines expert's caliber and computational algorithms, is expected to offer an immensely quick but analytical based method of scanning data. The complete system is, therefore, promising to reform the traditional approach to conduct a literature review. For humanities and social sciences, this method provided a structured approach to the conventional qualitative and hermeneutical scholarship. In this article, entrepreneurship research was used as an example. However, the approach can also be useful to review various aspects in other fields such as arts, cultural, or media studies, all depending on the user's customization.

## Appendix Supplementary materials

Image, application 1
